# Young Men, Mental Health, and Technology: Implications for Service Design and Delivery in the Digital Age

**DOI:** 10.2196/jmir.2291

**Published:** 2012-11-22

**Authors:** Louise A Ellis, Philippa Collin, Tracey A Davenport, Patrick J Hurley, Jane M Burns, Ian B Hickie

**Affiliations:** ^1^Brain and Mind Research InstituteFaculty of MedicineUniversity of SydneySydneyAustralia; ^2^Institute for Culture and SocietyUniversity of Western SydneySydneyAustralia; ^3^Young and Well Cooporative Research CentreMelbourneAustralia; ^4^Inspire FoundationSydneyAustralia; ^5^Orygen Youth Health Research CentreCentre for Youth Mental HealthUniversity of MelbourneMelbourneAustralia

**Keywords:** Young men, mental health, technology, service design and delivery, digital age, Internet use, games

## Abstract

**Background:**

Young men are particularly vulnerable to suicide, drug, and alcohol problems and yet fail to seek appropriate help. An alternative or adjunct to face-to-face services has emerged with widespread uptake of the Internet and related communication technologies, yet very little evidence exists that examines the capacity of the Internet to engage young men and promote help seeking.

**Objective:**

To explore young people’s attitudes and behaviors in relation to mental health and technology use. The aim was to identify key gender differences to inform the development of online mental health interventions for young men.

**Methods:**

A cross-sectional online survey of 1038 young people (aged 16 to 24 years) was used.

**Results:**

Young men are more likely than young women to play computer games, access online video/music content, and visit online forums. More than half of young men and women reported that they sought help for a problem online, and the majority were satisfied with the help they received. Significant gender differences were identified in relation to how young people would respond to a friend in need, with young men being less likely than young women to confront the issue directly.

**Conclusions:**

Online interventions for young men need to be action-oriented, informed by young men’s views and everyday technology practices, and leverage the important role that peers play in the help-seeking process.

## Introduction

Young men in Australia have higher rates of completed suicide, antisocial behavior, and alcohol or other substance misuse problems than young women [1,2]. They are also less likely to seek help with only 13% of young men aged 16 to 24 years seeking help when experiencing a mental health difficulty compared with 31% of young women [3].

The factors associated with poorer help-seeking practices in young men are complex [4]. Research suggests that young men have poorer mental health knowledge and higher mental health stigma than young women [5,6]. Additionally, Western norms of masculinity can function as a barrier to effective help-seeking, with males feeling social pressure to be self-reliant, suppress emotions, and manage their personal problems independently [7,8]. In order to promote help-seeking behavior for young men, it is now being argued that innovative improvements must be made to the design and delivery of mental health services to ensure that they are relevant and meet the unique needs of this demographic [4,8-10].

An alternative or adjunct to face-to-face services has emerged with widespread uptake of the Internet and related communication technologies, such as mobile phones, game consoles, and tablet computers [11]. These information and communications technologies (ICTs) are ideally suited to accommodate young men’s preferences for autonomous and anonymous channels for seeking help [12,13]. Studies of free access online programs designed to improve mental health outcomes (eg, MoodGYM, Reach Out Central) have shown promise in trials with young people [14-16]. However, these studies have also highlighted the difficulty and importance of sustained engagement (particularly for young males) if results are to be maximized [16,17]. This represents a key challenge for self-directed Internet-based programs. Both the academic and policy literature has identified the urgent need to develop evidence-based mental health strategies that target young men and leverage their ICT use to promote help-seeking [11,12,18]. Although considerable research exists regarding barriers to help-seeking and stigma among young people, more needs to be known about their ICT use and help-seeking preferences [9]. Specifically, we must understand how young men and women differ in their attitudes and behaviors in relation to mental health and ICT use if we are to develop new methods that are specifically tailored to meet their needs and expectations [19]. Here, we report the findings from a national online survey that explored young people’s attitudes and behaviors towards mental health, online habits, and technology use, as well as their experiences of using the Internet for information, help, or support.

## Methods

### Survey Design and Sample

A survey was administered online for a three-month period, from January 25 to March 27, 2010. Recruitment was achieved via snowball sampling, leveraging online social networking services. Online sampling was used as a way of reaching young people who are normally difficult to access via random-digit dialing or panel methods, and as a way of reducing social desirability effects [20]. Following recent trends in recruitment [21], an advertisement was placed on Facebook, a popular online social networking site, and participants were encouraged to promote the survey to their peers, who then completed the survey and further promoted the study through their networks. The Facebook advertisement was specifically targeted to appear on the pages of Australian Facebook users between the ages of 16 to 24 years. The advertisement consisted of a short title (“mental health and technology”), an image, and a longer description (“tell us what you think about how technology might be used to encourage young people to engage with mental health services”. A total of 1484 people clicked on the Facebook advertisement (the average cost per click was $0.42), and individuals who chose to participate were taken to the online questionnaire, which was hosted on the third-party website SurveyMonkey. The survey was also specifically advertised through youth-serving organizations, including youth centers and clinics, online service providers, charities, colleges, universities, and relevant government organizations, via a flyer and link to the survey, which was distributed via email.

This study had ethics approval from the University of Sydney Human Research Ethics Committee (Protocol No. 11209). Participants gave consent online and understood that their participation was voluntary, confidential, and non-identifiable. No incentives were offered for taking part. Participants included 1038 young people aged 16 to 24 years.

### Survey Measures

#### Technology Use

Two key questions were developed to examine young people’s use of a range of technologies. The first question asked about their use of various ICTs (eg, computers, PlayStation, Facebook, multiplayer games). The second question asked about their frequency of use of various types of media and entertainment (eg, newspapers, Internet for music, Internet for social networking, television) over the previous three months. Respondents rated this question on a six-point Likert scale (1 = “everyday” to 6 = “never”).

#### Attitudes and Behaviors Towards Mental Health

Two sets of questions were selected from the *headspace* community survey [9]. The first set of questions asked respondents what they would do if they thought a friend might be experiencing a mental health problem (eg, “keep out of their way”, “tell them to just get over it”, “try to include them in social activities with other friends”), with items being rated on a five-point Likert scale (1 = “very likely” to 5 = “very unlikely”). The second set of questions asked respondents whether they have ever talked about their problems on the Internet (“yes/no”), and if so, whether chatting with other people via the Internet helped (“yes/no”). Finally, respondents were asked how satisfied they were with the information/support they received on the Internet (1 = “very dissatisfied” to 4 = “very satisfied”).

#### Psychological Distress

The six-item Kessler Psychological Distress Scale (K6) [22] assesses the frequency with which an individual experiences symptoms of general psychological distress such as nervousness, tiredness, hopelessness, and restlessness on a five-point Likert scale (0 = “none of the time” to 4 = “all of the time”). Scores are then summed to create a total score that ranges from zero to 24.

#### Technology and Mental Health

A set of questions was developed to measure preferences for receiving mental health information and support through technology including: accessing a website with information and/or factsheets; a website with a question and answer service that sends short message service (SMS) or emails; a website with online clinic; interactive single-player games teaching life skills; and interactive multiplayer games teaching life skills.

### Data Analysis

The survey data were analyzed using the Statistical Package for the Social Sciences (SPSS) version 20.0. All surveys, both completed and uncompleted, were analyzed. Differences between young men and women were assessed using either chi-square analysis for nominal and ordinal dependent variables with two to four categories or analysis of variance (ANOVA) for ordinal dependent variables with five or more categories. A *P* value < .05 was considered statistically significant. For a select number of questions, we examined the results separately for the total sample and the psychological distressed sample (ie, those whose K6 scores were equal to or greater than 13) [23].

## Results

### Survey Participants

A total of 1038 young people (aged 16 to 24 years) participated in the survey (53.2% female; n = 552/1038; mean age = 18.84 years; SD age = 2.75). The majority of participants provided complete data (completion rate = 71.9%). The survey had good national coverage with respondents from all Australian States and Territories. 41.9% (n = 432/1030) were from New South Wales and Australian Capital Territory; 24.6% (n = 254/1030) were from Victoria and Tasmania; 18.1% (n = 186/1030) were from Queensland; and 15.3% (n = 158/1030) were from Western Australia, South Australia, and the Northern Territory. Seventeen people (2.2%; n = 17/790) identified themselves as Aboriginal and/or Torres Strait Islander origin; and 21.1% (n = 168/797) spoke a language other than English at home. Most of the sample were in full-time study at school, Technical and Further Education (TAFE) or university (63.7%, n = 475/746), while 16.0% (n = 119/746) were employed full-time (30 or more hours per week), and 8.4% (n = 63/746) were employed part-time (less than 30 hours per week).

### Young People’s Interests and Technology Use

The vast majority of respondents used mobile phones (98.0%, n = 878/896), iPod/MP3 players (89.7%, n = 802/894) and computers (desktops, 77.1%, n = 691/896; laptops, 84.0%, n = 752/895). Facebook was by far the most popular social networking website (92.7%, n = 831/896), with only 37.6% (n = 337/896) of the sample using MySpace and only 22.1% (n = 198/896) using Twitter. As shown in Table 1, the most noticeable gender differences were for game play, with significantly more males playing single-player, multiplayer, and interactive games than females. Other significant differences were found with more males accessing video websites and forums, and more females visiting Facebook and information sites.

**Table 1:** Gender differences in ICT use; N = 896^a^.

	Males (yes) n (%)	Females (yes) n (%)	χ^2^
N	403	493	
Mobile phone	388 (96.3)	490 (99.4)	10.92^b^
Facebook	363 (90.1)	468 (94.9)	7.77^b^
iPod/MP3 player	355 (88.5)	447 (90.7)	1.10
Laptop computer	328 (81.6)	424 (86.0)	3.21
Information websites	320 (79.4)	426 (86.4)	7.81^b^
Landline phone	321 (80.0)	394 (79.9)	0.00
Desktop computer	323 (80.1)	368 (74.6)	3.81
MSN	310 (76.9)	359 (72.8)	1.97
Video websites	343 (85.1)	321 (65.1)	46.22^c^
Forums	250 (62.0)	255 (51.7)	9.58^b^
Interactive games	249 (61.8)	237 (48.1)	16.80^c^
Nintendo/Wii	201 (49.9)	223 (45.2)	1.92
Single-player games	268 (66.5)	137 (27.8)	134.16^c^
PlayStation	213 (53.0)	191 (38.7)	18.14^c^
MySpace	151 (37.5)	186 (37.7)	0.01
Xbox	205 (51.0)	117 (23.7)	71.45^c^
Multiplayer games	220 (54.6)	99 (20.1)	115.18^c^
Bulletin boards	158 (39.2)	157 (31.8)	5.27^d^
Skype	148 (36.7)	148 (30.0)	4.51^d^
Twitter	83 (20.6)	115 (23.3)	0.96
Bebo	53 (13.2)	69 (14.0)	0.13

^a^ Denominators vary due to missing data. Rows are ordered according to frequency of endorsement.

^b^ *P* < .01.

^c^ *P* < .001.

^d^ *P* < .05.

Respondents were also asked more broadly about the kinds of media and entertainment they use. The most popular forms of media and entertainment across the sample were the Internet for social networking or communicating (74.1% use it daily, n = 657/887), the Internet for music (62.6% use it daily, n = 556/888), and the Internet for general information (58.2% use it daily, n = 516/886). Internet use was significantly more popular than watching TV (52.7% use it daily, n = 470/892) or listening to the radio (36.1% use it daily, n = 318/881). However, as shown in Table 2, males used the Internet to access games and music more frequently than females. Males also played games on a console or computer far more frequently than females. On the other hand, females listened to the radio and went to the cinema more frequently than males.

**Table 2:** Gender differences in media and entertainment use; N = 892^a^.

Level of usage (1 = “everyday” to 6 = “never”)	Males M (SD)	Females M (SD)	F
Internet for social networking or communicating	1.54 (1.19)	1.44 (0.96)	1.75
Internet for general information	1.70 (1.19)	1.68 (0.96)	0.07
Internet for music	1.58 (1.13)	1.80 (1.19)	7.55^b^
TV	1.98 (1.38)	1.81 (1.24)	3.64
Radio	2.76 (1.74)	2.44 (1.63)	8.00^b^
DVDs or videos	3.00 (1.27)	3.11 (1.21)	1.59
Newspapers	3.15 (1.63)	3.03 (1.43)	1.30
Games on a console or computer	2.62 (1.66)	3.91 (1.75)	125.26^c^
Internet for games	3.06 (1.83)	3.74 (1.86)	29.84^c^
Magazines	3.95 (1.43)	3.88 (1.26)	0.54
Cinema	4.14 (0.93)	4.27 (0.87)	4.92^d^

^a^ Rows are ordered according to frequency of endorsement.

^b^ *P* < .01.

^c^ *P* < .001.

^d^ *P* < .05.

### Young People’s Attitudes and Behaviors Towards Mental Health

Respondents were asked what they would do in the next few days if they noticed a friend was going through a tough time. The vast majority of the sample reported that they would be “likely” or “very likely” to listen to their friend and try to help them work out what to do (94.8%, n = 886/934), encourage them to focus on the positive things in life (88.3%, n = 825/934), and try to include them in social activities with other friends (87.5%, n = 812/928). However, as shown in Table 3, there were significant gender differences on most items with males being more likely than females to: “tell them to just get over it”; “tell them about their own worries to help them put their problems in perspective”; and “keep out of their way to give them some space”. On the other hand, females were significantly more likely than males to report that they would: “listen to them and try to help them work out what to do”; “try to include them in social activities with other friends”; “talk with someone else who knows them well about what to do”; and “encourage them to focus on the positive things in life”.

**Table 3:** Gender differences in what young people would do for a friend experiencing a mental health problem; N = 934.

Likelihood (1 = “very likely”; 5 = “very unlikely”)	Males M (SD)	Females M (SD)	F
Keep out of their way to give them some space	2.92 (1.00)	3.04 (0.84)	4.03^a^
Listen to them and try to help them work out what to do	1.71 (0.75)	1.43 (0.61)	40.32^b^
Encourage them to focus on the positive things in life	1.84 (0.88)	1.65 (0.78)	12.82^b^
Tell them about your worries to help them put their own problems in perspective	2.73 (1.08)	2.98 (1.02)	13.14^b^
Tell them that things will improve soon	2.20 (0.99)	2.21 (1.00)	0.00
Tell them about others who have got over similar problems	2.65 (1.08)	2.53 (1.06)	3.35
Tell them to just get over it	3.62 (0.86)	3.91 (0.49)	42.39^b^
Try to include them in social activities with other friends	2.04 (1.02)	1.70 (0.80)	31.03^b^
Encourage them to avoid situations that might upset them	2.15 (1.06)	2.07 (1.02)	1.42
Talk with someone else who knows them well about what to do	2.17 (1.12)	1.89 (0.96)	16.59^b^

^a^ *P* < .05.

^a^ *P* < .001.

The most “likely” or “very likely” sources of help respondents would suggest to a friend with a mental health problem would be: friends (88.0%, n = 816/927); a counselor (75.5%, n = 703/931); doctor (74.9%, n = 698/932); family member (68.5%, n = 637/930); and websites (45.9%, n = 422/920). They would be least likely to recommend posters or pamphlets, a church leader, teacher, or community member or center. However, there were significant gender differences on all variables apart from friends, with males being less likely to recommend each of the sources of help than females (see Table 4).

**Table 4:** Gender differences in sources of help young people would suggest to a friend with a mental health problem; N = 931^a^.

Likelihood (1 = “very likely”; 5 = “very unlikely”)	Males M (SD)	Females M (SD)	F
Friends	1.78 (0.86)	1.70 (0.81)	2.20
Counselor	2.21 (1.07)	1.89 (0.98)	22.08^b^
Doctor	2.25 (1.05)	1.87 (0.88)	35.21^b^
Family	2.33 (1.11)	2.08 (1.02)	12.52^b^
Websites	2.72 (1.02)	2.52 (1.02)	8.18^c^
Telephone helplines	2.89 (1.05)	2.55 (1.02)	25.35^b^
Community center	3.04 (0.98)	2.72 (1.09)	22.11^b^
Trusted community member	2.99 (0.96)	2.78 (0.98)	10.68^c^
Teacher	3.04 (0.98)	2.75 (0.94)	20.47^b^
Posters or pamphlets	3.16 (0.89)	2.94 (0.92)	12.70^b^
Church leader	3.26 (1.05)	3.08 (1.05)	6.80^c^

^a^ Rows are ordered according to frequency of endorsement.

^b^ *P* < .001.

^c^ *P* < .01.

### Young People’s Internet Use for Information, Help, or Support

The average time respondents spend using the Internet each day was 4.7 hours (4.4 hours for females and 5.2 hours for males). Significantly more males than females used the Internet after 11 p.m. at night (75.4% compared with 62.1%; χ^2^=17.53, *P* < .001), and significantly more females than males had talked about their problems online (62.1% females vs. 54.9% males; χ^2^ = 4.53, *P* = .020). Most females said that talking online “helped” (81.9%, n = 245/299) and that they were “satisfied” or “very satisfied” with the online help they received (85.1%, n = 256/301). Similarly, most males said that talking online “helped” (81.3%, n = 169/208) and that they were “satisfied” or “very satisfied” with the online help they received (82.9%, n = 174/210).

Of the total sample whose K6 scores were indicative of psychological distress (males: n = 130; females: n = 189), 69.4% (n = 221/318) said they had sought help for their problems online. Again, significantly more females than males had talked about their problems online (73.5% of females vs. 63.6% of males; χ^2^=3.60, *P* = .038). However, a similar proportion of females and males reported that talking online “helped” (79.0% compared with 71.6%; χ^2^=1.54, *P* = .141).

The survey also asked participants to indicate their preferences for receiving mental health information and support through technology (see Tables 5 and 6). The top two responses for males and females across the total sample were: website with information and/or fact sheets (males: 48.1%, n = 234/486; females: 59.6%, n =329/552) and website with online clinic (males: 38.5%, n = 187/486; females: 48.7%, n = 269/552). Similarly, the top two responses within psychologically distressed sample were: website with information and/or fact sheets (males: 62.3%, n = 81/130; females: 70.4%, n = 133/189) and website with online clinic (males: 51.5%, n = 67/130; females: 56.1%, n = 106/189).

**Table 5:** Gender differences in preferences for receiving mental health information and support through technology; Total sample^a^.

	Males n (%)	Females n (%)	χ^2^
N	486	552	
Website with information and factsheets	234 (48.1)	329 (59.6)	13.66^b^
Website with online clinic	187 (38.5)	269 (48.7)	11.03^c^
Website with question and answer service	140 (28.8)	230 (41.7)	18.63^b^
Website promoting well-being	118 (24.3)	243 (44.0)	44.41^b^
Website with multimedia content	144 (29.6)	165 (29.9)	0.01
Interactive single-player game teaching life skills	87 (17.9)	145 (26.3)	10.43^c^
Interactive multiplayer game teaching life skills	88 (18.1)	119 (21.6)	1.93

^a^ Denominators vary with missing data. Rows are ordered according to frequency of endorsement.

^b^ *P* < .001.

^c^ *P* < .01.

**Table 6:** Gender differences in preferences for receiving mental health information and support through technology; Psychologically distressed sample^a^.

	Males n (%)	Females n (%)	χ^2^
N	130	189	
Website with information and factsheets	81 (62.3)	133 (70.4)	2.27
Website with online clinic	67 (51.5)	106 (56.1)	0.64
Website with question and answer service	55 (42.3)	92 (48.7)	1.26
Website promoting well-being	36 (27.7)	90 (47.6)	12.80^b^
Website with multimedia content	50 (38.5)	60 (31.7)	1.54
Interactive single-player game teaching life skills	29 (22.3)	59 (31.2)	3.06
Interactive multiplayer game teaching life skills	28 (21.5)	47 (24.9)	0.48

^a^ Denominators vary with missing data. Rows are ordered according to frequency of endorsement.

^b^ *P* < .001.

## Discussion

Despite considerable investment in mental health over the last decade, there has been no shift in national data on young men’s help-seeking [3]. The results of this survey clearly signal that ICT plays a central role in young people’s lives and may present an alternative or adjunct solution. The young men in this study used ICT predominantly for entertainment and socializing, but they also used the Internet to find information and support. We found that more than half of all males and two-thirds of psychologically distressed males used the Internet for help-seeking. Furthermore, the males in this study reported high levels of satisfaction with their online help-seeking experiences. These results provide evidence for further investment into ICT-based mental health service provision—particularly to increase engagement with young men.

The data provide important information on young men’s ICT preferences and use. They were drawn to websites with video/music content and were far more likely than young women to play computer games. These insights suggest that the challenge of relevancy and engagement faced by mental health services may be addressed by action-oriented (rather than information or talk-based) strategies that target young men [24]. These strategies must be user-driven [10] and informed by young men’s views and everyday technology practices.

Importantly, the results highlight gender differences in how young people would respond to a friend who is experiencing a mental health problem. In comparison to females who would be more likely to respond proactively and intervene, young men stated that they would be less likely to confront the issue directly. This is consistent with previous research showing that young men have limited experience relative to young women in helping someone with an emotional concern and would be less likely to offer advice [5]. Thus, online strategies that encourage and empower males to help their friends with emotional concerns should be explored, especially given the important role that peers play in the help-seeking process [10].

### Limitations

Three potential limitations should be considered when interpreting the results of this study. First, given that we recruited participants using online methods, our sample was limited to young people with Internet access. Nevertheless, Internet access and use in Australia is very high: 97% of young people have personal access to the Internet [25], and the results of this study concerning ICT use are comparable with previous research [26,27]. Secondly, our sample appears to be skewed towards respondents with higher levels of psychological distress than we would expect from the general population. While our results may thus not be generalizable to the entire population, our success in recruiting young people with potential mental health issues is noteworthy and suggests that online snowball sampling techniques may be a particularly effective way to recruit young people with mental health issues to research—a group typically very hard to recruit. Thirdly, we cannot be certain that the observed gender differences are a result of gender per se or are the result of other factors, such as poor mental health knowledge or higher mental health stigma [5,6]. Future research aimed at exploring gender differences while controlling for these other factors will help elucidate this issue and give us a more comprehensive understanding of how to engage young men.

### Conclusion

Despite these limitations, the results of this study present a compelling argument for investment in ICT-based mental health initiatives that target young men. Investment should increase the visibility of already known and trusted youth mental health services as well as support the development of new interventions. The results suggest that further development of online mental health services that can respond effectively to young men’s questions with direct links to experts. Furthermore, online services that include the principles of gaming and music/video content, as well as provide opportunities to seek information and support autonomously and anonymously, could be particularly appealing to young men, although further research in this area is warranted. Social networking technology may also provide a powerful tool to promote social connectedness of young men that can support help-seeking and warrants further research. Finally, this survey was unable to account for the recent and rapid proliferation and popularity of tablets, smart phones, and mobile phone applications [28]. Further research should pay particular attention to the role of mobile media and applications in the delivery of strategies to promote help-seeking in young men.
